# The diagnostic accuracy of Sudoscan in TTR-FAP

**DOI:** 10.1186/1750-1172-10-S1-P42

**Published:** 2015-11-02

**Authors:** Jose Castro, Isabel Conceicao, Isabel Castro, Mamede de Carvalho

**Affiliations:** 1 Centro Hospitalar Lisboa Norte - Hospital de Santa Maria, Department of Neurosciences, 1600, Lisboa, Portugal; 2 Instituto de Medicina Molecular, Faculty of Medicine, Translational and Clinical Physiology Unit, 1600, Lisboa, Portugal

## Introduction

Small-fibre length-dependent sensory-motor and autonomic neuropathy is the hallmark of TTR-FAP.

SUDOSCAN was recently introduced as a quick and non-invasive method that measures electrochemical skin conductance (ESC) of palmar and plantar surfaces, through reverse iontophoresis. It has been described as a promising diagnostic tool in distal symmetric polyneuropathies, such as diabetic small fibre neuropathy.

## Objective

To evaluate the diagnostic accuracy of Sudoscan in patients with TTR-FAP.

## Methods

Forty stage I TTR-FAP patients were compared with 70 TTR-FAP asymptomatic carriers and 37 healthy controls, matched for age, gender and body-mass index. Inclusion criteria for TTR-FAP patients included normal sural nerve sensory action potential amplitude and plantar sympathetic skin response (SSR). Patients with diabetes were excluded. All subjects were assessed with Sudoscan in hands and feet, bilaterally.

## Results

Feet ESC was significantly reduced in Stage I patients compared with asymptomatic carriers and controls (57.8 ± 24.3 vs 76.5 ± 7.8 and 79.7 ± 5.1; p < 0.000). Hands ESC did not show significant difference between groups.

Receiver operating characteristic curve analysis revealed an area under the curve of 0.80 for the plantar ESC.

A significant correlation was found between plantar and Sural nerve action potential amplitude (0.320; p < 0.001).

## Conclusion

Sudoscan seems to be a promising diagnostic tool in TTR-FAP patients with normal conventional nerve conduction studies and preserved plantar SSR. However, its predictive value is unknown.

